# Detecting the contagion effect in mass killings; a constructive example of the statistical advantages of unbinned likelihood methods

**DOI:** 10.1371/journal.pone.0196863

**Published:** 2018-05-09

**Authors:** Sherry Towers, Anuj Mubayi, Carlos Castillo-Chavez

**Affiliations:** Arizona State University, Tempe, AZ, United States of America; Tulane University School of Public Health and Tropical Medicine, UNITED STATES

## Abstract

**Background:**

When attempting to statistically distinguish between a null and an alternative hypothesis, many researchers in the life and social sciences turn to binned statistical analysis methods, or methods that are simply based on the moments of a distribution (such as the mean, and variance). These methods have the advantage of simplicity of implementation, and simplicity of explanation.

However, when null and alternative hypotheses manifest themselves in subtle differences in patterns in the data, binned analysis methods may be insensitive to these differences, and researchers may erroneously fail to reject the null hypothesis when in fact more sensitive statistical analysis methods might produce a different result when the null hypothesis is actually false.

Here, with a focus on two recent conflicting studies of contagion in mass killings as instructive examples, we discuss how the use of unbinned likelihood methods makes optimal use of the information in the data; a fact that has been long known in statistical theory, but perhaps is not as widely appreciated amongst general researchers in the life and social sciences.

**Methods:**

In 2015, Towers *et al* published a paper that quantified the long-suspected contagion effect in mass killings. However, in 2017, Lankford & Tomek subsequently published a paper, based upon the same data, that claimed to contradict the results of the earlier study. The former used unbinned likelihood methods, and the latter used binned methods, and comparison of distribution moments.

Using these analyses, we also discuss how visualization of the data can aid in determination of the most appropriate statistical analysis methods to distinguish between a null and alternate hypothesis. We also discuss the importance of assessment of the robustness of analysis results to methodological assumptions made (for example, arbitrary choices of number of bins and bin widths when using binned methods); an issue that is widely overlooked in the literature, but is critical to analysis reproducibility and robustness.

**Conclusions:**

When an analysis cannot distinguish between a null and alternate hypothesis, care must be taken to ensure that the analysis methodology itself maximizes the use of information in the data that can distinguish between the two hypotheses.

The use of binned methods by Lankford & Tomek (2017), that examined how many mass killings fell within a 14 day window from a previous mass killing, substantially reduced the sensitivity of their analysis to contagion effects. The unbinned likelihood methods used by Towers *et al* (2015) did not suffer from this problem.

While a binned analysis might be favorable for simplicity and clarity of presentation, unbinned likelihood methods are preferable when effects might be somewhat subtle.

## Introduction

Binned statistical analysis methods, such as analysis methods based on histograms, are often used in analyses in the life and social sciences to distinguish between null and alternate hypotheses. Oftentimes, null and alternate hypotheses would manifest themselves in quite different patterns in the data, and despite the loss of information inherent in binned methods, the binned methodology is able to statistically distinguish between the two hypotheses. When this is the case, binned methods have the advantage of simplicity of execution, and clarity of presentation.

However, when differences are more subtle, binned methods may erroneously lead researchers to fail to reject the null hypothesis, even though there may be enough information in the unbinned data to support a different conclusion.

The benefits of using unbinned likelihood fits to increase the statistical power of an analysis when comparing null and alternate hypotheses are perhaps somewhat under-appreciated in the literature in the life and social sciences. The term “likelihood” was introduced by Fisher in 1925 [1], and is a mathematical expression that quantifies the joint probability of observing a particular data set, given a particular statistical hypothesis. The likelihood expression typically depends on one or more parameters, and the “best fit” parameter estimates are the values of the parameters that maximize the likelihood of observing the data, given the particular statistical model. Unlike binned data analysis methods, which necessitate loss of information upon binning, unbinnned likelihood methods have been shown to yield the smallest possible variance of estimators for large data sets; because the estimators have the smallest possible variance, they are thus maximally sensitive to potential effects of interest, and thus maximize the statistical power of the analysis [2–4]. Likelihood ratio tests can be used to compare two nested statistical models to determine if a more complicated “alternate” model is significantly more likely than a less complicated “null” model [2, 5].

As an example of the advantages of unbinned likelihood methods in increasing the statistical power of an analysis, here we compare and contrast two recent analyses of contagion in mass killings in America, both of which were based on exactly the same data, but used different methodology. One concluded that there was evidence of contagion in mass killings [6], while the later analysis contradicted this claim [7].

Copycat effects have long been suspected as playing a role in the incidence of mass killings [8–10]. However, the first quantification of this effect came in 2015, when Towers *et al* published an analysis of the temporal patterns in mass killings and school shootings in the U.S., and found significant evidence of contagion [6]. Under the hypothesis that a mass killing temporarily raises the probability of a similar event occurring in the near future, with an exponential decay in that probability, in Towers *et al* (2015) we found that each mass killing appears to inspire approximately 0.28 new mass killings ([0.10, 0.56], 95% CI), with an average decay period of the exponential of approximately 13 days.

Using the same data presented in Towers *et al* (2015), Lankford & Tomek (2017) published a subsequent analysis that claimed to find no significant evidence of contagion in mass killings [7]. They primarily based their conclusion on a simple analysis of how many events occurred within 14 days of a prior event, under the null hypothesis assumption that the data were randomly Uniformly distributed in time. They also compared the mean and variance of the distribution to the mean and variance expected under their null hypothesis, and performed statistical tests of the null hypothesis using these quantities with Students t, F, and Z tests.

The primary difference between the analysis of Lankford & Tomek (2017) and Towers *et al* (2015) is that the former used a simple binned analysis methodology (binning the data into only two bins), whereas the latter employed unbinned maximum likelihood methods to test for contagion effects (Beyond the necessary binning of the data into integer days, because for most reports of mass killings, the time of day was not noted). It is somewhat rare to find in the literature two analyses based on exactly the same data that come to differing conclusions when testing essentially the same hypotheses. The differing results of the analyses of Towers *et al* (2015) and Lankford & Tomek (2017) are thus an excellent example of the advantages of unbinned likelihood methods in optimizing the statistical power of an analysis.

In the following sections, we compare and contrast the methodology used by both analyses, and discuss in detail why unbinned likelihood methods provide the best sensitivity to distinguish between null and alternate analysis hypotheses. We also discuss how visualization of data can help guide the decision on which analysis methodologies might be most appropriate to distinguish between a null and alternate hypothesis.

In addition, we also discuss the issue of assessment of analysis robustness to methodological assumptions, like, for example, somewhat arbitrary data selections (over other, equally reasonable selections), or arbitrary selections in data binning when using binned methods. This is widely overlooked in practice in the life and social sciences, yet has serious implications for reproducibility and robustness of conclusions.

## Methods and materials

### Data

#### USA Today mass killing data

Data on mass killings in the U.S. between 2006 to the time of writing of this manuscript in October 2017 were obtained from a USA Today study that examined Federal Bureau of Investigation (FBI) data from the FBI Supplemental Homicide Reports, and hundreds of media reports and police documents to compile a list of incidents that involved four or more people being killed, not including the killer (data are available from, and described at, masskillings.usatoday.com, accessed October 2017). The USA Today study did not rely solely upon the FBI data, in part because the FBI data are based on voluntary reports by local police agencies and are thus an incomplete tally of mass murders in the U.S., and also because the data were found to only have a 61% accuracy rate when compared with data available from local police documents, apparently largely due to mistakes in transcription. For more details about the USA Today data set, see www.usatoday.com/story/news/nation/2013/12/03/fbi-mass-killing-data-inaccurate/3666953/ (accessed October, 2017).

The data used by the Towers *et al* (2015) and Lankford & Tomek (2017) analyses were the USA Today data between 2006 to 2013, which consisted of 232 events. The data are available online as part of the Towers *et al* (2015) publication [6] (https://doi.org/10.1371/journal.pone.0117259.s002, accessed October, 2017). Towers *et al* found that the incidents showed no significant dependence on month of the year, but were significantly more likely to occur on Saturdays (Pearson *χ*^2^ *p* = 0.04).

From 2014 to October, 2017, as reported by USA Today at masskillings.usatoday.com, there have been 102 additional mass killings.

### Modeling and statistical methods

#### Towers *et al* (2015) self-excitation contagion model and likelihood fitting methods

In a self-excitation contagion model, recent prior events increase the probability of another event happening in the near future, that then decays in time. In the Towers *et al* (2015) analysis, we employed an Exponential probability distribution to simulate the temporary rise and decay in this probability. Under the hypothesis that past events incite future events, the increased probability of an event occurring during the 24 hours of day *t*_*j*_ due to prior event that occurred on day *t*_*i*_ is thus [6]

$$\begin{array}{c} P\left(t_{j}|t_{i},T_{\text{excite}}\right)=\int_{t_{j}-t_{i}-1}^{t_{j}-t_{i}}dx\;\frac{e^{-x/T_{\text{excite}}}}{T_{\text{excite}}}, \end{array}$$ (1)

where *T*_excite_ is the average duration of the contagion process. This yields

$$\begin{array}{c} P\left(t_{j}|t_{i},T_{\text{excite}}\right)=e^{-\left(t_{j}-t_{i}-1\right)/T_{\text{excite}}}-e^{-\left(t_{j}-t_{i}\right)/T_{\text{excite}}}. \end{array}$$ (2)

In Towers *et al* (2015), we considered a self-excitation contagion model with an additional baseline (i.e.; non-contagion related) average number of events per day of *N*_0_(*t*). Taking into account all prior events in some stochastic data realization, the total number of expected events, *N*^exp^, on day *t*_*n*_ for that realization is thus

$$\begin{array}{c} N^{\text{exp}}\left(t_{n}|N_{\text{secondary}},T_{\text{excite}}\right)=N_{0}\left(t_{n}\right)+N_{\text{secondary}}\sum_{\forall t_{i}<t_{n}}P\left(t_{n}|t_{i},T_{\text{excite}}\right), \end{array}$$ (3)

where the summation is over all prior events. The parameters of this contagion model are the average number of secondary events inspired by the contagion of a single event, *N*_secondary_, the decay constant of the the Exponential contagion process, *T*_excite_, and whatever parameters are needed to describe the temporal evolution of the baseline number of events, *N*_0_(*t*). For instance, one can assume *N*_0_(*t*) is a constant, or a straight line with a slope. One can also use a non-parametric approach where *N*_0_(*t*) is calculated using a weighted running mean of the data itself; this latter option is preferable, because it takes into account potential longer term temporal trends in the data.

The functional form of *N*_0_(*t*) can also incorporate additional weights if needed in order to take into account day-of-week or seasonal effects, and indeed, in Towers *et al* (2015) we incorporated such weights when it was evident that there was significant evidence of dependence on season or day-of-week in a particular data set. For example, the USA Today mass killing data set showed no significant seasonality, but did show significant dependence on day-of-week.

In addition to specifying a functional form for the predicted model, in a likelihood analysis one must assume a probability distribution that describes the stochasticity in the data. In this case, the number of events observed per day is count data, thus the data are likely to be Poisson distributed, or Negative Binomially distributed if over-dispersed compared to a Poisson hypothesis [11].

The Poisson probability of observing *k* events when λ are predicted is

$$\begin{array}{ccc} p(k|\lambda ) & = & e^{-\lambda }\frac{\lambda^{k}}{k!}, \end{array}$$ (4)

where the prediction λ may be a complex model that depends on various parameters. In our case, λ = *N*^exp^, and *k* = *N*^obs^. If a total of *M* events are observed, *k*_*i*_ (*i* = 1, …, *M*), each with model prediction, λ_*i*_, the likelihood of observing the data given the model is [12]

$$\begin{array}{ccc} L & = & \prod_{i=i}^{M}e^{-\lambda_{i}}\frac{\lambda_{i}^{k}}{k_{i}!}. \end{array}$$ (5)

The best-fit parameters of the model are the ones that maximize this likelihood. Equivalently, because products of probabilities can rapidly lead to underflows when calculating the likelihood in practice, one finds the parameters that maximize the log-likelihood

$$\begin{array}{ccc} \log L & = & \sum_{i=i}^{M}[-\lambda_{i}+k\log\lambda_{i}-\log k_{i}!]. \end{array}$$ (6)

Maximizing the log-likelihood is equivalent to minimizing the negative log-likelihood, which is what is done in statistical analysis software for optimization. Note that the log *k*_*i*_! term depends only on the data, not the model parameters, thus in practice it is usually dropped from the Poisson log-likelihood expression to reduce computational complexity.

To estimate the values of *N*_secondary_ and *T*_excite_, we thus found the values of *T*_excite_ and *N*_secondary_ that minimized the Poisson negative log likelihood

$$\begin{array}{c} -\log L=\sum_{i=1}^{M}[N^{\text{exp}}\left(t_{i}|N_{\text{secondary}},T_{\text{excite}}\right)-N_{i}^{\text{obs}}\log N^{\text{exp}}\left(t_{i}|N_{\text{secondary}},T_{\text{excite}}\right)], \end{array}$$ (7)

where *M* is the number of days spanned by the data, $N_{i}^{\text{obs}}$ is the number of observed events on the *i*^th^ day ($N_{i}^{\text{obs}}\geq 0$). The constraint is applied that $\sum_{i=1}^{M}N^{\text{exp}}\left(t_{i}|N_{\text{secondary}},T_{\text{excite}}\right)=\sum_{i=1}^{M}N^{\text{obs}}$. Beyond the necessary binning by day of the data because most incident reports do not include the time-of-day at which an incident occurred, this was an unbinned likelihood fit; the number of observed events on each and every separate day informed the fit for the parameters, even when the number of observed events was zero.

The null hypothesis model simply consisted of *N*^exp^ = *N*_0_. In order to determine if the full model was significantly more likely than the null model, we used the Wilks likelihood ratio test [5] to determine if the negative log likelihood of the former model was significantly smaller than that of the latter.

In Towers *et al* (2015), we cross-checked the robustness of the results to the various assumptions of the analysis, including the period over which the running mean, *N*_0_(*t*), was calculated, and also the use of Negative Binomial likelihood over the Poisson likelihood.

#### Lankford & Tomek (2017) uniform null hypothesis model

In the analysis of Lankford & Tomek (2017), they considered a null hypothesis model that assumed the data were Uniformly distributed in time. Note that the assumption that data are Uniformly distributed in time is equivalent to assuming that the time difference between events is Exponentially distributed.

Lankford & Tomek did not fit an alternate hypothesis contagion model for the values of the decay constant of the Exponential contagion process, *T*_excite_, nor the number of secondary events incited by contagion, *N*_secondary_. Instead, they based their analysis on simple methods to test if the time difference between events in the data were apparently consistent with their Exponential null hypothesis.

In their methodology, they assessed the mean and variance in the observed time difference between events, and compared the results to those expected from the Exponential distribution. Unfortunately, as we will see, only using the first two moments of a distribution ignores the fact that two probability distributions can have very different shapes, yet still have the same mean and variance. In comparing the mean and variance of the data to that expected from an Exponential distribution, Lankford & Tomek did not test whether or not an alternate hypothesis was significantly more likely than the null hypothesis, but rather simply tested, based on these two quantities, whether or not the null hypothesis was rejected.

In addition to this, Lankford & Tomek (2017), also examined what fraction of events occurred within a nominal cutoff of two weeks after the prior events, and compared this to the fraction expected from Exponentially distributed data, in effect binning the data into only two bins. Again, they did not test whether a specific alternate model was more likely, but simply tested whether or not the null hypothesis was supported. Lankford & Tomek did not assess the robustness of their analysis results to other assumptions of this time cutoff, or indeed to the use more finely binned data.

#### Visualization of simulated data under the Towers *et al* (2015) and Lankford & Tomek (2017) models

Visualization of the predictions of two models can help to elucidate differences between them, and help to guide choices in analysis methodology that would be most sensitive to these differences.

**Fig 1:**
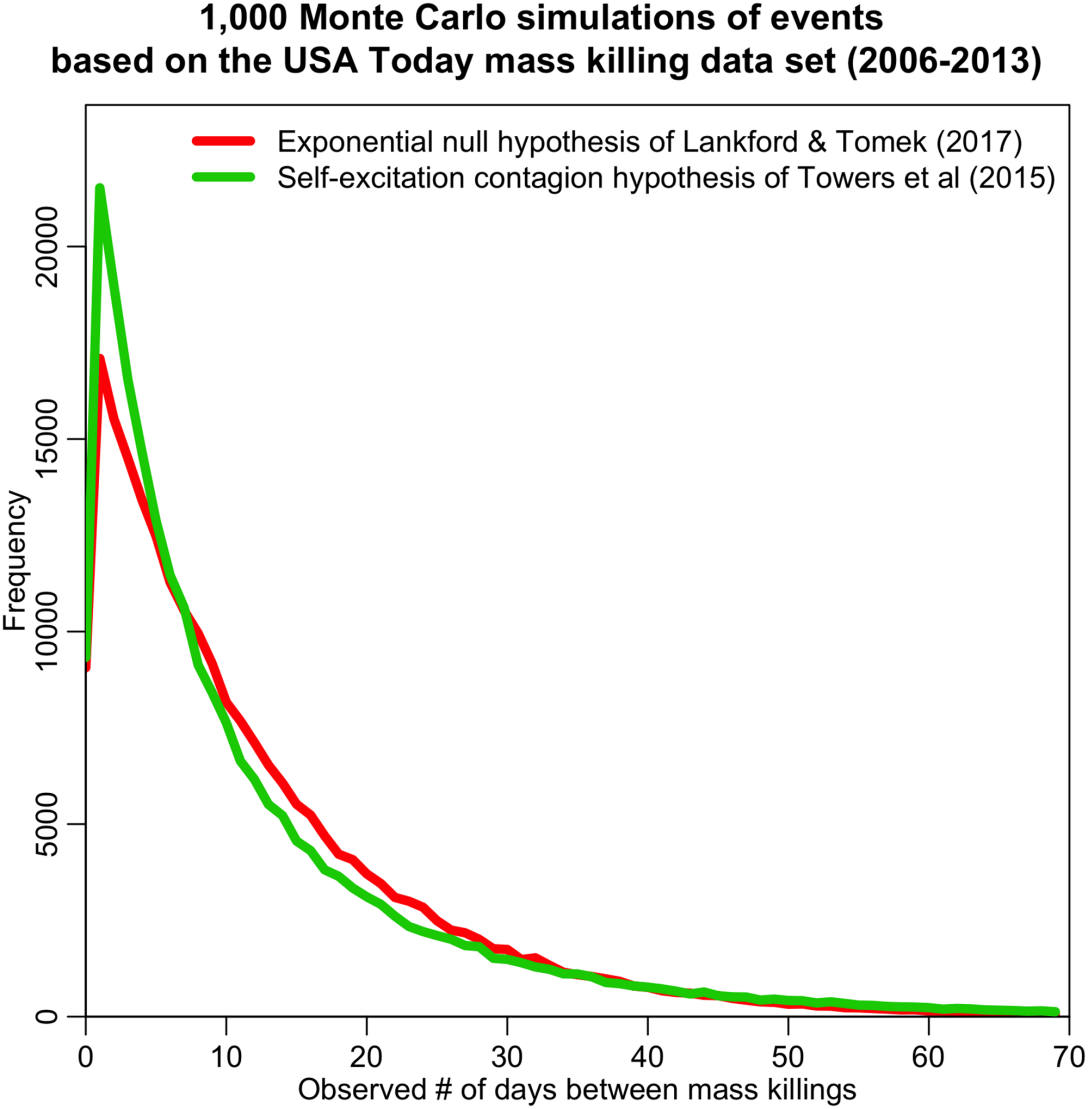
The time difference between events, based on 1,000 Monte Carlo simulations of the eight year span of the USA Today mass killing data set. In red is the prediction of the Exponential null hypothesis model of Lankford & Tomek (2017), and in green is the self-excitation contagion model of Towers *et al* (2015). These simulated data highlight the difference in shapes of the distributions hypothesized in the two analyses. Despite the differences in shape, however, the means of the two distributions are the same, and the fraction of events occurring within 14 days of a previous event is only 2% different.

The expected distribution of time-between-events for the Towers *et al* (2015) contagion model and Lankford & Tomek (2017) null hypothesis model is shown in Fig 1, under the assumption that the data are binned in integer days. The visualization is based on 1,000 Monte Carlo simulations of data over an eight year time span, based on the patterns observed in the eight years of data in the USA Today mass killing data set between 2006 to 2013.

In the case of simulation based on the Lankford & Tomek analysis, the time between events is randomly drawn from an Exponential distribution with the same average rate as observed in the USA Today mass killing data set. The temporal distance between events is then binned in integer days. Events that occur on the same day are assigned a temporal distance of zero. The details of the Monte Carlo simulation of the Towers *et al* contagion model are fully described in Reference [6] and the supplementary information therein. Briefly; to simulate a self-excitation process under some hypothesis of *N*_secondary_ and *T*_excite_, along with a functional parameterization of *N*_0_(*t*) obtained from the long-term running mean of the data, we start with 0 events on day *t*_1_ and calculate the expected number of events on the next day *N*_exp_(*t*_2_) using Eq 3. The number of events on day *t*_2_ is then simulated with a random number drawn from the Poisson distribution with mean *N*_exp_(*t*_2_). On each subsequent day, *t*_*i*_, the number of events depends on the timing of the past events, and is simulated with a random number drawn from the Poisson distribution with mean *N*_exp_(*t_i_*). This is repeated for the desired length of the time series.

As seen in Fig 1, compared to the Exponential null hypothesis model of Lankford & Tomek (2017), the contagion model of Towers *et al* (2015) predicts that an excess of events will occur in the few days after a mass killing, followed by a relative deficit of events after around one week. It is notable that even though the shapes of these two distributions are quite different, by the 14 day cutoff used by the Lankford & Tomek analysis the excess and deficit of events before and after around 7 days begin to cancel each other out; the Exponential hypothesis of Lankford and Tomek predicts that around 69% of events will fall within 14 days, whereas the contagion model of Towers *et al* predicts that 71% fall within that time period.

The average means of the simulations of the two distributions are the same (12.5 days). The 1,000 simulations of the null model of Lankford & Tomek (2017) have an average variance of 156.5, with 95% CI [105.3, 225.7]. The 1,000 simulations of the contagion model of Towers *et al* (2015) have an average variance of 212.9, with 95% CI [138.6, 347.8]. Note that the two 95% confidence intervals overlap the entire region between the two averages; this means that any observed variance between the averages obtained from the two models would be statistically consistent with either model.

Thus, based on this visualization, we can see that even though the distribution shapes are distinctly different, the binned methods of Lankford & Tomek would likely be unable to statistically distinguish between a null hypothesis and contagion model hypothesis for modest data sample sizes of only a few hundred events. Nor would the mean and variance likely be useful. In the following section, we show the comparison of the models to the data to determine if this was indeed the case.

## Results

**Fig 2:**
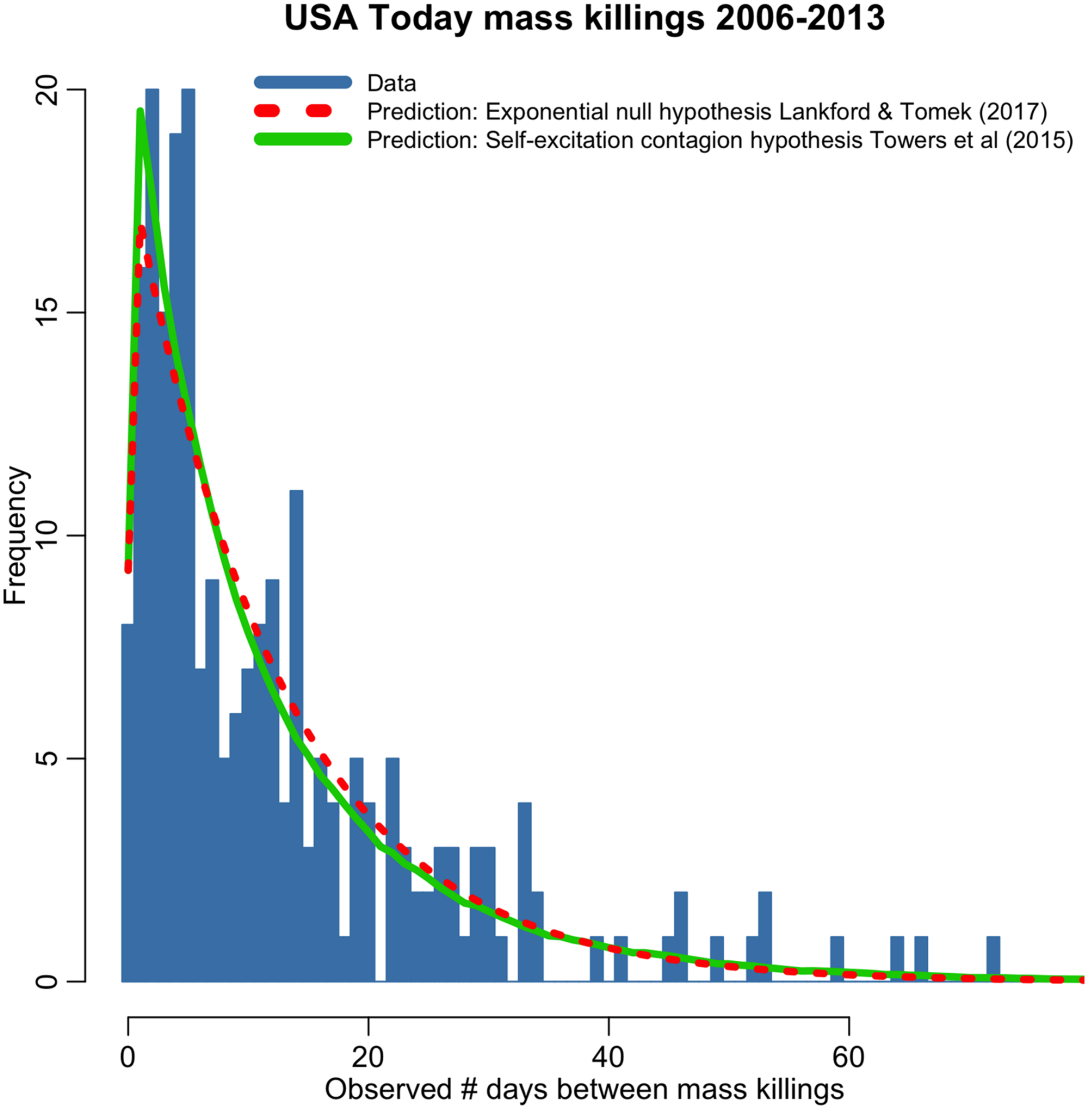
Distribution of the number of days between events in the USA Today 2006–2013 mass killing sample, binned into integer days. Overlaid in red is the prediction of the Lankford & Tomek (2017) Exponential null hypothesis model. Overlaid in green is the prediction of the Towers *et al* (2015) contagion model. While both models have similar predicted means and variances, and give similar predictions for the fraction of events occurring within 14 days, the contagion model does a significantly better job of describing the excess of events occurring a few days after a mass killing.

In Fig 2 we show the distribution of the number of days between events in the USA Today mass killing sample between 2006 to 2013 (N = 232). Overlaid in red is the Exponential null hypothesis distribution of Lankford & Tomek (2017), and overlaid in green is the self-excitation contagion model of Towers *et al* (2015). In the few days immediately after a mass killing, there is an excess of events observed in the data compared to the predictions of the Lankford & Tomek null model. The Towers *et al* self-excitation contagion model does a better job of describing this phenomenon. Indeed, based on this distribution that is binned into integer days, the contagion model of Towers *et al* (2015) is significantly more likely than the Lankford & Tomek (2017) null model; the Aikike Information Criterion (AIC) statistic for the Lankford & Tomek model, assuming a Negative Binomial likelihood to account for over-dispersion in the data, is 272.3, and the AIC of the Towers *et al* model is 260.8.

In contrast, the methods used by Lankford & Tomek (2017) are not sensitive to the differences in the models; the mean of the observed time differences in the data is 12.5 days, as is the mean of both the Lankford & Tomek and Towers *et al* hypothesized distributions. The variance of the time differences in the data is 176, with a predicted variance of 157 from the Lankford & Tomek null hypothesis, and 213 from the Towers *et al* contagion hypothesis. The Lankford & Tomek model predicts that 69% of the data will fall within 14 days of a prior mass shooting, while the Towers *et al* contagion model predicts 71%. The observed fraction is 71%. Both the Lankford & Tomek (2017) and Towers *et al* (2015) models give statistically consistent predictions for all of these quantities.

**Fig 3:**
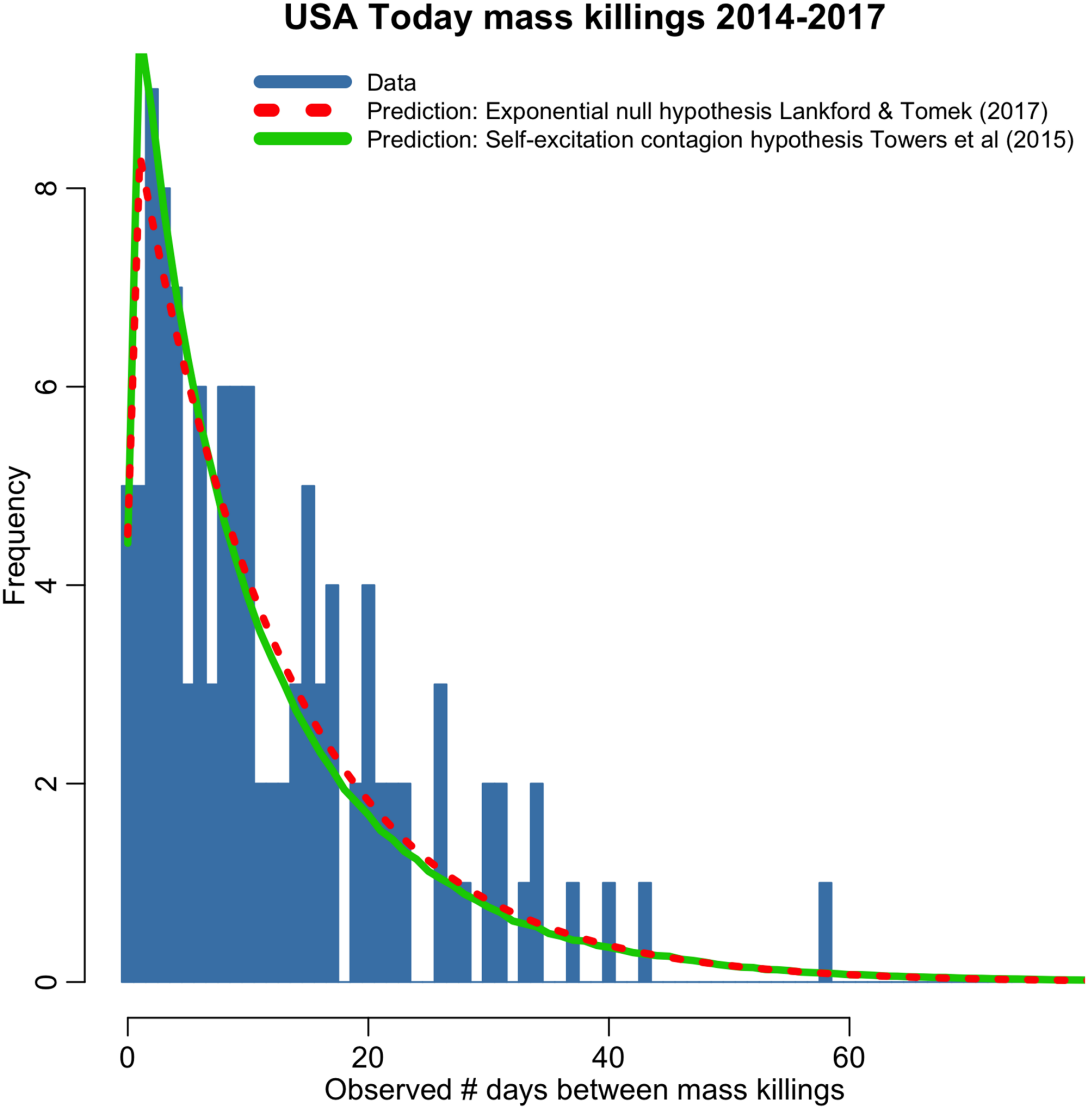
Distribution of the number of days between events in the USA Today 2014 to October 2017 mass killing sample, binned into integer days. Overlaid in red is the prediction of the Lankford & Tomek (2017) Exponential null hypothesis model, as fit to the 2006–2013 data and extrapolated to this sample. Overlaid in green is the extrapolated prediction of the Towers *et al* (2015) contagion model. The contagion model again does a significantly better job of describing the excess of events occurring a few days after a mass killing.

Up to the time of writing of this manuscript in October 2017, there have been over 100 mass killings since the 2006 to 2013 data examined in the Lankford & Tomek and Towers *et al* analyses. This provides an excellent opportunity to test the predictive power of the extrapolation of the Lankford & Tomek null model, and the Towers *et al* contagion model. In Fig 3 we show the distribution of the number of days between events in the USA Today mass killing sample from 2014 to October 2017 (N = 102). Overlaid in red is the Exponential null hypothesis distribution of Lankford & Tomek (2017), and overlaid in green is the self-excitation contagion model of Towers *et al* (2015). Both models were fit to the 2006 to 2013 USA Today sample, and then extrapolated to the new data. In the new data set, we again see evidence of an elevated number of events occurring within a few days of each mass killing, which is again better described by the Towers *et al* model; the AIC statistic for the Lankford & Tomek null model, assuming a Negative Binomial likelihood to account for over-dispersion in the data, is 324.9, and the AIC of the Towers *et al* contagion model is 313.3.

For this new data, we again find that the methods used by Lankford & Tomek (2017) are insensitive to the differences between the models. The mean and variance of the observed time differences in this independent data are 13.4 and 148, respectively, and the fraction falling within 14 days of a prior event is 62%. Both the Lankford & Tomek (2017) and Towers *et al* (2015) models give statistically consistent predictions for all of these quantities.

## Discussion

Use of unbinned likelihood analysis methods to maximize analysis sensitivity have been common for at least two decades in the fields of astronomy and physics (see for example, References [2, 4, 13–18]). In both fields, interesting “signal” events are almost overwhelmingly buried in a background of uninteresting “background” events, necessitating the use of more sophisticated methodologies to maximize analysis sensitivity.

Unbinned likelihood analyses have also been employed in analyses of animal movement patterns [19, 20], but in general the author has not noted widespread use of the methods in the life sciences, or, in particular, the social sciences.

**Fig 4:**
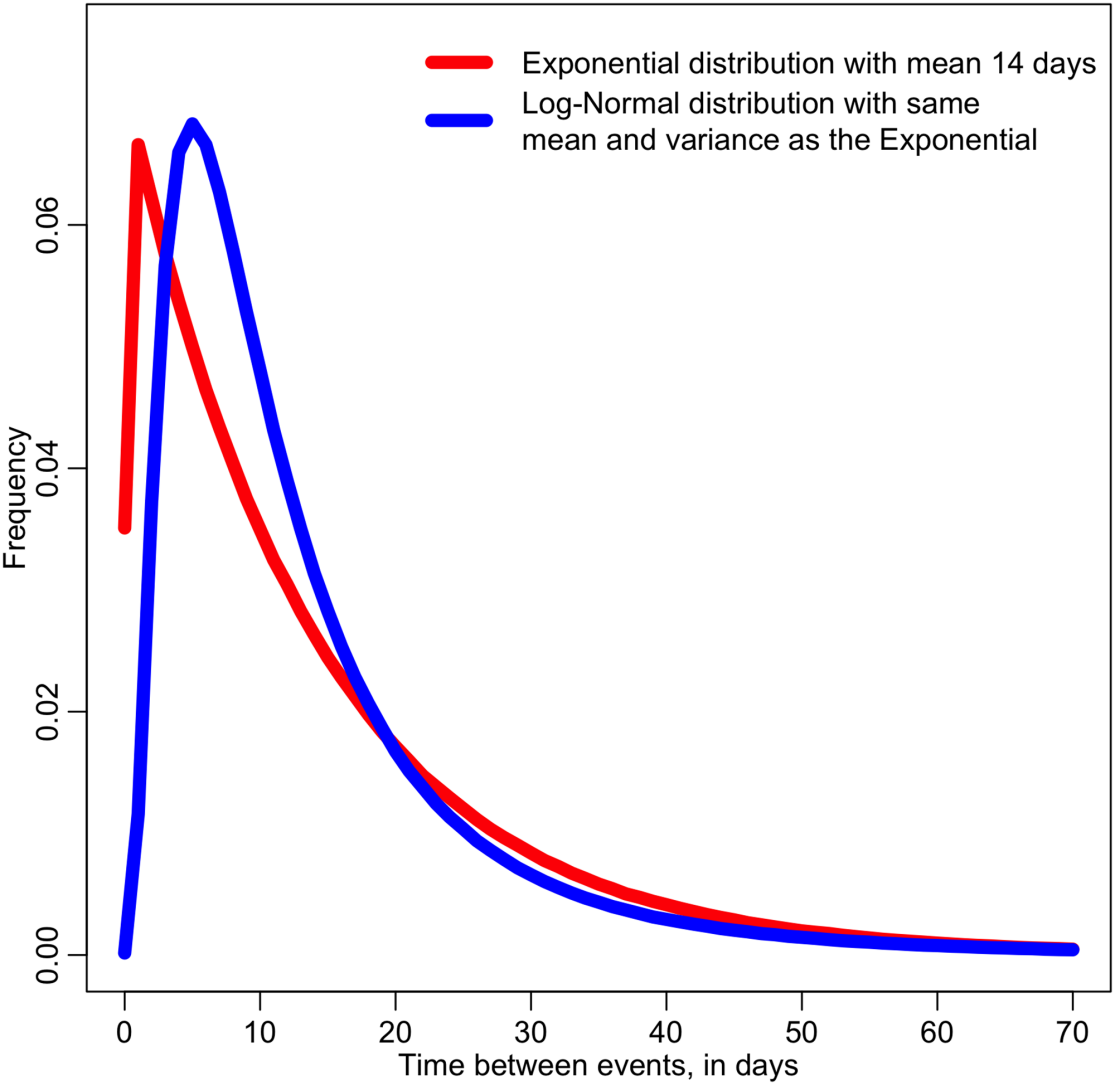
Illustrative example showing how two distributions can have exactly the same mean and variance, but can be quite different in shape. In red is the Exponential distribution with mean and variance equal to 14 days, and in blue is the Log-Normal distribution with exactly the same mean and variance. This underlines why simply testing the mean and variance of a distribution is not necessarily sensitive to differing distribution hypotheses. In addition, even though the shapes of these particular distributions are quite different, the fraction of events falling within 14 days happens to be quite similar; 65% for the Exponential, and 68% for the Log-Normal. This demonstrates how coarse binning of data reduces sensitivity to the shape of distributions.

In our comparison of two recent analyses of contagion in mass killings, we noted that the Lankford & Tomek (2017) analysis made several simplistic assumptions in methodology that served to decrease the sensitivity of the analysis compared to the unbinned likelihood methods used by Towers *et al* (2015). For example, Lankford & Tomek examined whether or not the null hypothesis of Uniformly distributed events in time (implying Exponentially distributed time between events) was supported by the mean and variance of the distribution of times between events. However, complex probability distributions can have exactly the same mean and variance, while having quite different shapes. We show an example of this in Fig 4; shown in red is the expected time between events when the data are Exponentially distributed with a mean of 14 days. Shown in blue is the Log-Normal distribution with exactly the same mean and variance as the Exponential distribution. The two distributions have distinctly different shapes, but simply testing the mean and variance would be insensitive to the differences between the distributions. However, an unbinned likelihood analysis of data comparing the two hypotheses would be maximally sensitive to the differing shapes, and even an analysis based on a moderately sized sample of a few hundred events would likely be sufficient to significantly favor one hypothesis over the other.

Our simulated data comparing the null hypothesis model of Lankford & Tomek to the self excitation contagion model of Towers *et al* is shown in Fig 1. When compared to the data, both models provided statistically consistent estimates of the mean and variance of the distribution. However, the predicted distributions of the models have distinctly different shapes, and, as seen in Fig 2, the Towers *et al* contagion model does a significantly better job of describing the excess of events in the few days after a mass killing.

Lankford & Tomek also examined the fraction of events that fall within 14 days of a mass killing. In effect, they binned the data into only two bins, and in doing so virtually all information about the shape of the distribution was lost. Because of this, both models provided statistically consistent estimates of the fraction of events that fall within 14 days. Visualization of the data with finer binning as shown in Fig 2, and an analysis of the AIC of the two models based on that distribution, shows that the data are indeed more consistent with the contagion model.

Further, the data used in the analyses of both Lankford & Tomek (2017) and Towers *et al* (2015) only spanned the period between 2006 to 2013. Since then, there have been over 100 more mass killings, providing an opportunity to both sets of researchers to test the predictive capability of their respective models. As we have shown in Fig 3 with the visualization of the distribution of the time between events in the data binned into integer days, and an analysis of the AIC of the two models based on that distribution, the extrapolation of the Towers *et al* contagion model does a significantly better job of predicting the temporal patterns in the subsequent data, compared with the Lankford & Tomek null model.

While the analysis of Lankford & Tomek (2017) made several simplistic assumptions, it should be noted that the analysis of Towers *et al* (2015) incorporated its own simplifying assumptions; we assumed an exponential decay in contagion immediately after an event occurred. But if indeed media coverage is the vector of contagion for these events, the temporal patterns in media coverage would affect the temporal distribution of the contagion component of the self-excitation model (causing a delay in the peak of the contagion probability distribution when it is convolved with the temporal trends in media coverage). Indeed, a slight delay of a day or two appears to be evident in the data distributions seen in Figs 2 and 3, so the data would appear to likely support such a hypothesis. Finding a method to quantify the temporal trends in media coverage, and incorporating such effects into an analysis, would be an interesting avenue of further inquiry, although it should be noted that it would dramatically increase the computational complexity of the analysis.

## Summary

We have shown that analyses that coarsely bin data, or test hypotheses based on only means and variances of a distribution, can be insensitive to differences between the distributions of two competing hypotheses. In contrast, unbinned likelihood methods make maximal use of the information in the data, resulting in estimators with the minimum possible variance [3]. When two competing hypotheses manifest themselves in more subtle patterns in the data, unbinned likelihood methods may be able to statistically distinguish between the two hypotheses, where binned methods might fail. While this is well known in statistical theory, in practice the potential pitfalls of using binned methodology may perhaps be somewhat under-appreciated in the life and social sciences.

These concepts were illustrated here in our comparison of two analyses of contagion in mass killings that have appeared in the literature; both of which used exactly the same data, but different analysis methodologies. The Towers *et al* (2015) analysis of mass killings used unbinned maximum likelihood methods to examine the temporal distribution of the events, and found significant evidence of contagion [6]. In contrast, the analysis of Lankford & Tomek (2017) examined exactly the same data using coarsely binned analysis methods, and found no evidence of contagion in mass killings, claiming to contradict the results of the earlier study [7]. The comparison of the two analyses provides an excellent example of the power of unbinned likelihood methods; the very coarsely binned method used by Lankford & Tomek was not sensitive to differences between a null hypothesis model of no contagion and an alternate hypothesis of a self-excitation contagion model.

We showed here that a simple visualization of the distribution of the difference in time between events in the mass killing data helped to elucidate the differences between the two competing models, and that the data distribution was significantly more consistent with the distribution expected from a contagion model, rather than the distribution expected from a null hypothesis model of no contagion.

It must be kept in mind that it is admirable to strive for simplicity of analysis methods. However, this must be balanced with analysis sensitivity. When there are very distinct differences in the manifestations of null and alternate hypotheses, simple binned methods may be sufficient to detect the significant differences, and manuscripts summarizing such analyses are comprehensible to researchers with even the most basic statistical expertise. However, with such analyses (or any analysis, for that matter) care must be taken to cross-check the robustness of the analysis results to all assumptions made in the methodology, including any assumptions of the binning used (both in the number of bins, and their widths) [21]. Unfortunately, in general such robustness cross-checks to methodology assumptions are too often neglected in the literature. It should be stressed here that, beyond this latter point, there was nothing wrong in the technical manifestation of the Lankford & Tomek analysis; the statistical tests they employed were appropriate to their methodology. The primary problem was that the statistical power of the simple methodology employed was severely impacted to the point that no inference could be made.

When simple methods cannot discern between two models, one must consider whether the analysis methodology being employed is sufficient to the task of making a comparison that maximizes the use of the information in the data. When the differences are subtle, unbinned likelihood methods are always preferable.
